# Characterization of the Activation of Protein Tyrosine Phosphatase, Receptor-Type, Z Polypeptide 1 (PTPRZ1) by Hypoxia Inducible Factor-2 Alpha

**DOI:** 10.1371/journal.pone.0009641

**Published:** 2010-03-10

**Authors:** Victoria Wang, David A. Davis, Ravindra P. Veeranna, Muzammel Haque, Robert Yarchoan

**Affiliations:** HIV and AIDS Malignancy Branch, Center for Cancer Research, National Cancer Institute, National Institutes of Health, Bethesda, Maryland, United States of America; Texas A&M University, United States of America

## Abstract

**Background:**

Hypoxia inducible factors (HIFs) are the principal means by which cells upregulate genes in response to hypoxia and certain other stresses. There are two major HIFs, HIF-1 and HIF-2. We previously found that certain genes are preferentially activated by HIF-2. One was protein tyrosine phosphatase, receptor-type, Z polypeptide 1 (PTPRZ1). PTPRZ1 is overexpressed in a number of tumors and has been implicated in glioblastoma pathogenesis.

**Methodology/Principal Findings:**

To understand the preferential activation of PTPRZ1 by HIF-2, we studied the PTPRZ1 promoter in HEK293T cells and Hep3B cells. Through deletion and mutational analysis, we identified the principal hypoxia response element. This element bound to both HIF-1 and HIF-2. We further identified a role for ELK1, an E26 transformation-specific (Ets) factor that can bind to HIF-2α but not HIF-1α, in the HIF-2 responsiveness. Knock-down experiments using siRNA to ELK1 decreased HIF-2 activation by over 50%. Also, a deletion mutation of one of the two Ets binding motifs located near the principal hypoxia response element similarly decreased activation of the PTPRZ1 promoter by HIF-2. Finally, chromatin immunoprecipitation assays showed binding of HIF and ELK1 to the PTPRZ1 promoter region.

**Conclusions/Significance:**

These results identify HIF-binding and Ets-binding motifs on the PTPRZ1 promoter and provide evidence that preferential activation of PTPRZ1 by HIF-2 results at least in part from cooperative binding of HIF-2 and ELK1 to nearby sites on the PTPRZ1 promoter region. These results may have implications in tumor pathogenesis and in understanding neurobiology, and may help inform the development of novel tumor therapy.

## Introduction

Oxidative metabolism is essential for many processes in mammalian cells, and cells must rapidly respond to the stress of reduced oxygen or face irreversible cell damage and death. Organisms respond to hypoxia through a number of adaptations, such as increased glycolysis, angiogenesis, and erythropoeisis [1]. Many of these adaptations are mediated through the activation of specific genes by hypoxia-inducible factors (HIFs) [2]. HIFs are heterodimers composed of either HIF-1α or HIF-2α combined with constitutively expressed HIF-1β to form HIF-1 and HIF-2, respectively [3]. HIF-1α and HIF-2α are constitutively expressed, but under normoxic conditions are hydroxylated at specific proline residues, resulting in ubiquitination through the interaction with von Hippel-Lindau factor protein (pVHL) and proteosomal degradation. Under hypoxic conditions, this proline hydroxylation is inhibited, leading to their rapid accumulation and binding to HIF-1β to form active HIFs. Other stimuli such as oxidative stress may also increase HIF levels [4], [5]. HIFs activate hypoxia-responsive genes by binding to specific sequences in their promoter regions called hypoxia-responsive elements (HRE); a number of HRE have been analyzed and the consensus sequence is 5′-RCGTG-3′, where R is A or G, although variants have been reported[4]. Most functional HRE also contain an adjacent element, termed the ancillary sequence.

There is emerging evidence that HIFs are important in tumorigenesis, in part through their ability to induce angiogenesis [6], [7], [8]. Moreover, von Hippel-Lindau (VHL) syndrome, an inherited disorder caused by a mutation in the pVHL gene that leads to elevated HIF levels, is associated with the development of a number of tumors, especially renal cell carcinoma or pheochromocytoma [9], [10].

A number of initial studies suggested that in general, HIF-1 and HIF-2 do not differ with regard to their target genes. However, more recent studies in our lab and elsewhere showed that certain genes are differentially activated by HIF-1 and HIF-2 [11], [12], [13], [14], [15], [16]. Studying these effects by microarray in HEK293T cells, we identified 56 genes that were upregulated by hypoxia and/or by HIFs [11]. Fourteen of these genes were preferentially activated by HIF-1 and 10 were preferentially activated by HIF-2. One HIF-2-specific gene was protein tyrosine phosphatase, receptor-type, z polypeptide 1 (PTPRZ1), also called receptor protein tyrosine phosphatase beta (RPTP-β). PTPRZ1 was upregulated about 6 fold by HIF-2α, but not by HIF-1α. HIF-2-specific induction of PTPRZ1 was also observed using real-time PCR. Interestingly, like most of the HIF-2-specific genes identified in this study, PTPRZ1 was only minimally upregulated by hypoxia. PTPRZ1 encodes for several additional proteins by variable splicing, including RPTPβ short form, phosphacan, and phosphacan short isoform [17], [18].

PTPRZ1 is preferentially expressed in the central nervous system, and has been shown to play a role in recovery from demylenating lesions [19]. Moreover, PTPRZ1 is overexpressed in a number of tumors, including hepatocarcinoma, renal carcinoma, and glioblastoma [20]. Its has been most studied in glioblastoma, where there is evidence that it plays a role in tumor pathogenesis and may be a target for therapy [20], [21], [22], [23]. However, relatively little is known about the mechanisms controlling PTPRZ1 expression. In this paper, we dissected the molecular basis for the upregulation of PTPRZ1 by HIF-2 and show that ELK1, a member of the Ets family of transcription factors, is involved in its regulation.

## Results

### Identification of Functional HREs in the PTPRZ1 Promoter

**Figure 1:**
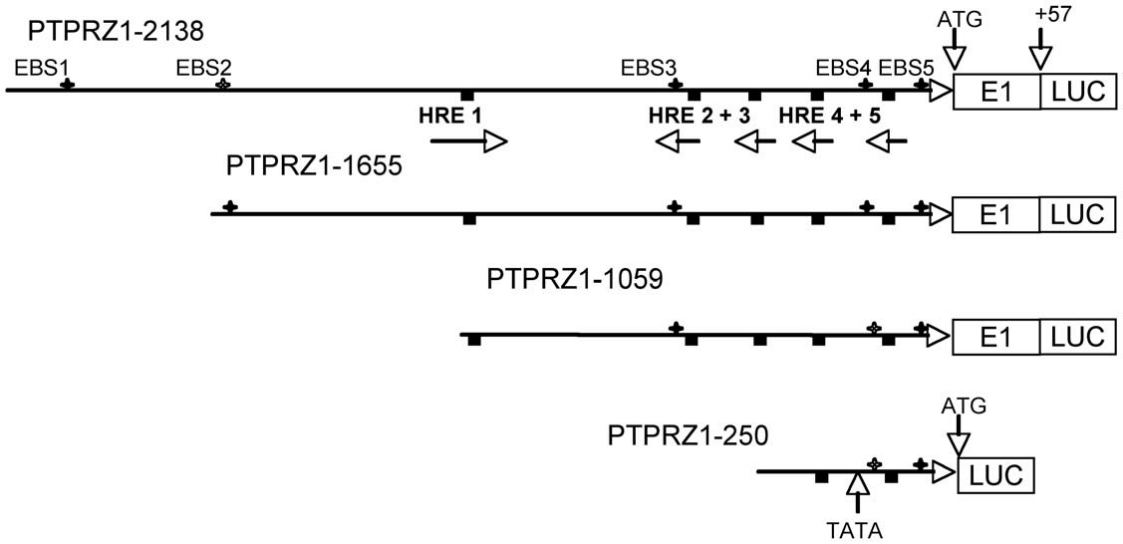
PTPRZ1 luciferase promoter constructs showing the location of the potential hypoxia response elements (HRE) and Ets binding sequences (EBS). Each HRE is denoted as a square and each EBS as a plus sign. The TATA box and the ATG start site are indicated in the PTPRZ1-250 promoter. Each promoter construct extends 57 bp into the PTPRZ1 coding region prior to the luciferase (LUC) sequence except for the PTPRZ1-250 promoter, which stops at the ATG. The HRE consensus sequences and direction of each HRE are also indicated. The core HRE sequences are: HRE1, CCGTG; HRE2, CACGC; HRE3, CACGC; HRE4, CACGCACG; HRE5, CACGG.

To investigate the molecular basis for the regulation of PTPRZ1 expression and in particular its upregulation by HIF-2, but not HIF-1, we analyzed the PTPRZ1 promoter sequence for HRE consensus sequences [4] and identified five potential HREs (Figure 1). The first, (HRE1) is encoded in 5′->3′ direction on the sense strand while the other four (HREs 2–5) are encoded in the 5′->3′ direction on the antisense strand (HRE1; -973-968, HRE2; -447-452, HRE3; -294-299, HRE4; -130-138, and HRE5; -25-30) (Figure 1). These HREs all contain the consensus core HRE motif RCGTG (or a CCGTG variant) on either the positive or negative strand [4]. Nearly all HREs have this consensus motif, although not all such sequences mediate up-regulation by HIFs [4], [24]. To study the role of these potential HREs, a PTPRZ1 luciferase reporter promoter construct (−2138 to +57) containing the five putative HREs was prepared. In addition, sequential deletion of this promoter was carried out yielding promoters of 1655 bp, 1059 bp, and 250 bp (Figure 1). The three longest promoters contain the first 57 base pairs of exon 1 of PTPRZ1, but this region was not retained in the 250 bp promoter.

**Figure 2:**
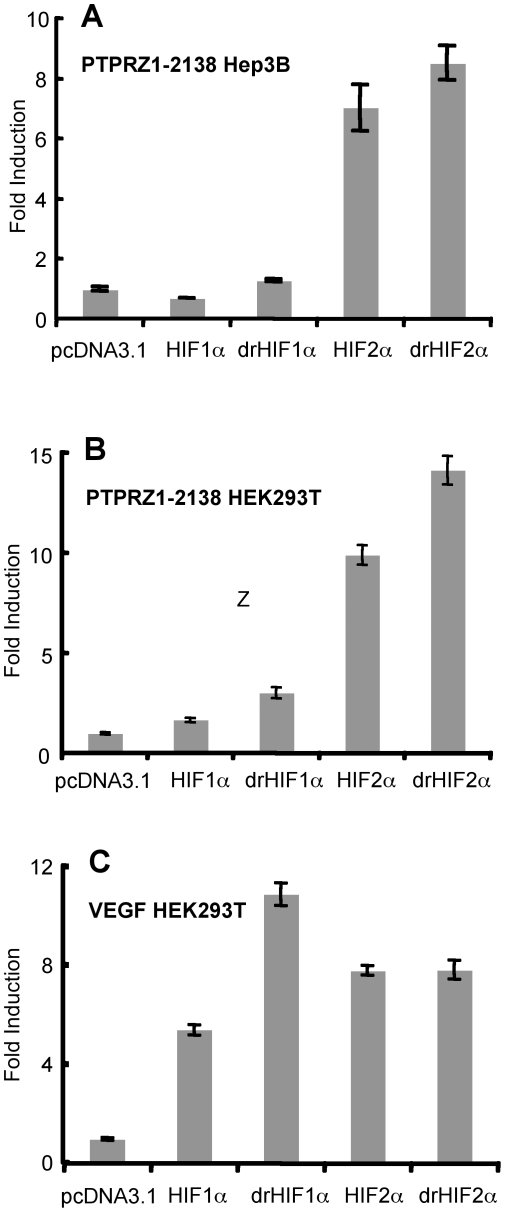
Comparison of the activation of the PTPRZ1-2138 luciferase reporter by HIF-1α and HIF-2α and their degradation-resistant forms. (A) Hep3B or (B) HEK293T cells were co-transfected with 300 ng of PTPRZ1-2138 promoter and 50 ng of an internal β-gal control plasmid in the presence of 250 ng of an expression plasmid encoding HIF-1α, drHIF-1α, HIF-2α, drHIF-2α, or pcDNA3.1 expression plasmid control. Values are expressed as fold increase over the reporter lacking expression vector (pcDNA3.1) and represent the mean of triplicate determinations in one representative experiment out of three with similar results. Error bars denote the standard deviations. (C) Results obtained with a VEGF luciferase reporter control plasmid under the same conditions.

The PTPRZ1 promoter constructs were transiently transfected into HEK293T or Hep3B cells along with plasmids encoding HIF-1α, HIF-2α, or their degradation-resistant forms (drHIF-1α or drHIF-2α). HIF accumulation in the nucleus of the cells following transfection with these plasmids was confirmed by Western blot analysis of nuclear extracts. Higher levels of HIF-1α and HIF-2α accumulated in these cells following transfection as compared to hypoxia and the levels were even greater when the degradation-resistant plasmids were utilized (results not shown). When the PTPRZ1-2138 promoter was cotransfected with plasmids encoding HIF-1α or drHIF-1α, there was little or no increase in promoter activity in either Hep3B or 293T cells (Figure 2). By contrast, co-transfection with HIF-2α or drHIF-2α increased the promoter activity 7-10 fold in Hep3B cells and 10–14 fold in HEK293T cells. Consistent with the results of our previous microarray study of HEK293T cells[11], little or no increase in PTPRZ1-2138 promoter activity was observed when the cells were exposed to hypoxia (results not shown). As a control, we carried out a similar experiment using the VEGF promoter, which is responsive to both HIF-1α and HIF-2α [11], [12], [25]. The VEGF promoter was upregulated 5 fold or more by HIF-1α, drHIF-1α, HIF-2α, and drHIF-2α (Figure 2C).

**Figure 3:**
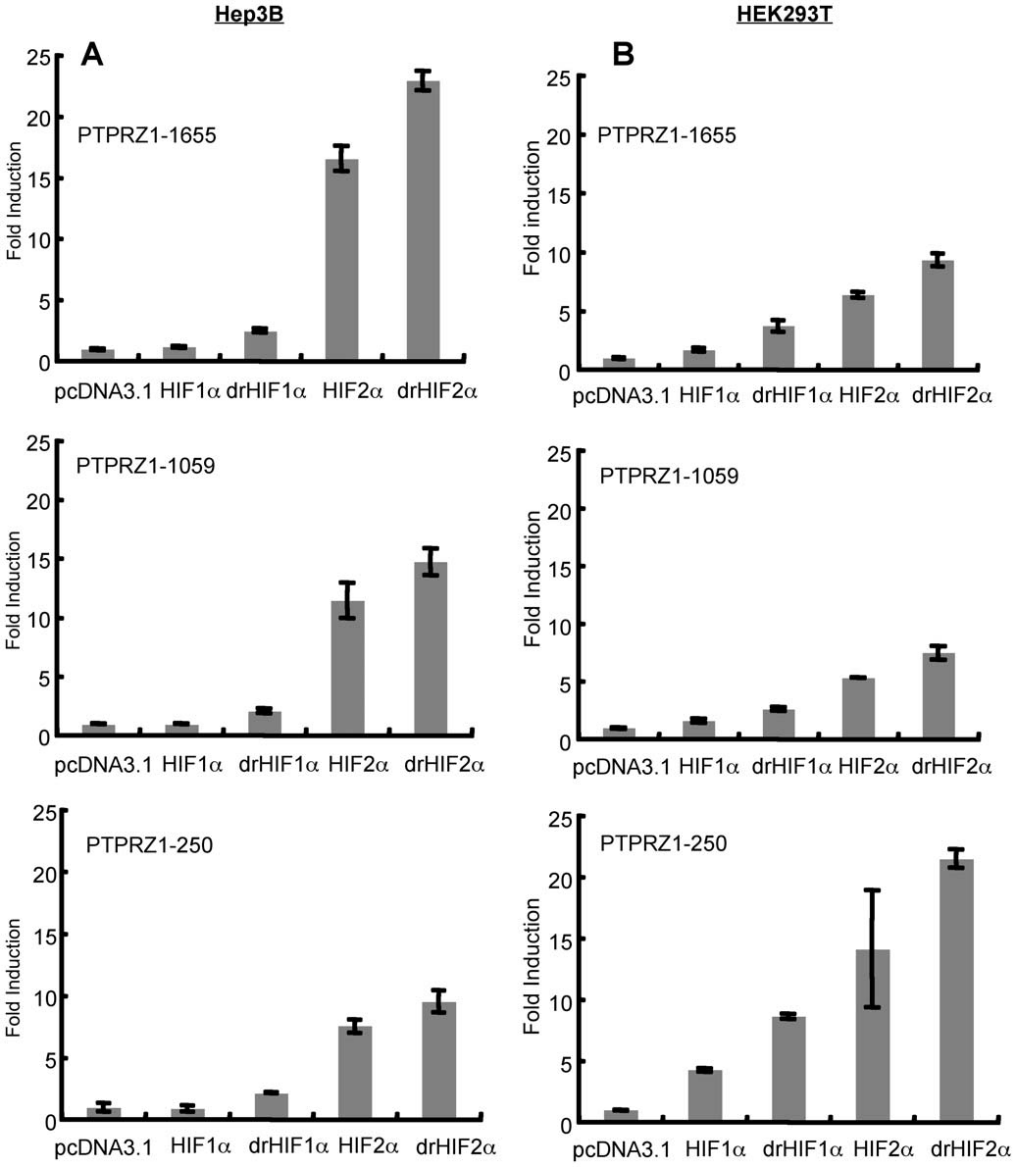
Comparison of the activation of truncated forms of PTPRZ1 luciferase reporter by HIF-1α and HIF-2α and their degradation-resistant forms. (A) Hep3B or (B) HEK293T cells were co-transfected with 300 ng of each PTPRZ1 promoter and 50 ng of an internal β-gal control plasmid in the presence of 250 ng of an expression plasmid encoding HIF-1α, drHIF-1α, HIF-2α, drHIF-2α, or pcDNA3.1control. Results are expressed as in Figure 2.

To narrow down the region of the PTPRZ1 promoter mediating HIF-2α-specific activation, experiments were carried out with truncated forms (PTPRZ1-1655, PTPRZ1-1059, PTPRZ1-250). Co-transfection of HIF-1α or drHIF-1α-encoding plasmids had little effect on these promoters in Hep3B cells (Figure 3A). By contrast, co-transfection with HIF-2α or drHIF-2α-encoding plasmids increased promoter activity in PTPRZ1-1655 by 23 fold, and upregulation was also seen with the shorter constructs (Figure 3A). Similarly, in HEK293T cells, all the promoters were activated to a greater degree by HIF-2α as compared to HIF-1α (Figure 3B), although the relative specificity of up-regulation by HIF-2α was not as clear cut. In both cell lines, the shortest PTPRZ1 promoter, PTPRZ1-250, was still preferentially up-regulated by HIF-2α, indicating that the sequences leading to preferential HIF-2α activation were contained within this region.

### Mutational Analysis of PTPRZ1-250 Promoter

**Figure 4:**
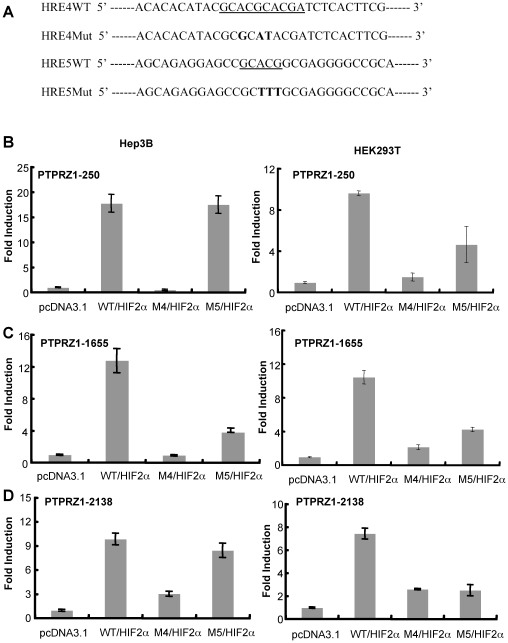
Effect of HRE4 and HRE5 mutations in the PTPRZ1 promoter on the response to HIF-2α. (A) DNA sequences for the HRE4 and HRE5 WT and mutant constructs. The underlined region indicates the location of the putative HREs, and bold letters indicate the mutations from WT for each mutant construct. (B–D) HEP-3B and HEK293T cells were co-transfected with plasmids encoding HIF-2α and either the wild-type reporter promoter (WT), the HRE4 mutant reporter promoter (M4), or the HRE5 mutant reporter promoter (M5). WT reporter cotransfected without HIF (pcDNA3.1) was used as a control. (B) PTPRZ1-250. (C) PTPRZ1-1655. (D) PTPRZ1-2138. Values were expressed as in Figure 2; shown are the mean and standard deviation of triplicate determinations.

The PTPRZ1-250 sequence contained two putative HREs: HRE4 and HRE5. To further identify the functional HRE(s) that mediated the response to HIF-2α, we performed site-directed mutagenesis of these two HREs. A 3 bp mutation of HRE4 or HRE5 was introduced into each promoter to generate PTPRZ1 mutants M4 and M5 respectively (Figure 4A). HRE4 is a near-tandem HRE, and the mutations were designed to affect both consensus sequences. Mutation of HRE5 did not affect PTPRZ1-250 promoter activity in Hep3B cells but led to a modest reduction of promoter activity in HEK293Tcells (Figure 4B). By contrast, mutation of HRE4 virtually eliminated HIF-2α-induced up-regulation of the PTPRZ1-250 promoter in both Hep3B and HEK293T cells (Figure 4B). In subsequent experiments utilizing larger promoter constructs, the HRE4 mutation also led to a greater loss in activity than the HRE5 mutation (Figures 4C and 4D). These results indicate that HRE4 is a main contributor to the response of the PTPRZ1 promoter, although there may also be a contribution from HRE5 as well as other potential HREs.

### Functional Binding of HIF to the PTPRZ1 Promoter

**Figure 5:**
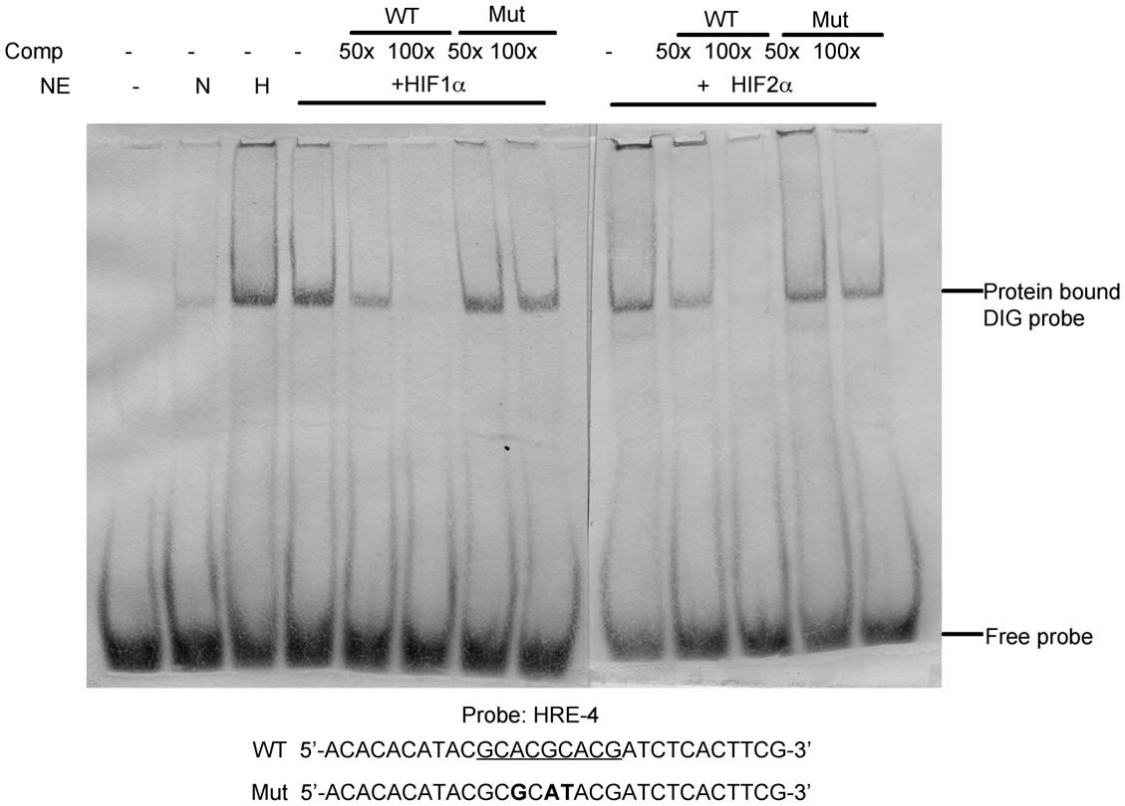
HIF-1 and HIF-2 bind to PTPRZ1 oligonucleotide probes containing HRE4. DIG-labeled synthetic oligonucleotide containing HRE4 was incubated with nuclear extracts (NE) from normoxic (N), 16 hour hypoxic (H) or HIF-transfected (HIF-1α or HIF-2α) HEK293T cells and analyzed on a non-denaturing polyacrylamide gel. Where indicated, unlabeled wild type (WT) or mutant (Mut) oligonucleotides (at 50x and 100x of the labeled probe) were added to the binding reaction. Protein-DNA complexes were separated, blotted to a nylon membrane, and probed with anti-digoxigenin antibody conjugated to alkaline phosphatase. Comp denotes unlabeled probe used for binding competition. The sequence of the probe and WT and Mut competing oligonucleotides used is shown at the bottom. WT HRE4 sequence is underlined, and nucleotide changes in the Mut sequence are shown in bold. The positions of the DIG-labeled HIF complexes and free probe are indicated with arrows.

To assess the binding of HIF-1 and HIF-2 to HRE4, electrophoretic mobility shift assays were performed using nuclear extracts from HEK293T cells exposed to normoxia, hypoxia, or transfected with HIF-1α or HIF-2α in normoxia (Figure 5). The DIG-labeled oligo probe containing HRE4 formed a complex with nuclear extracts from hypoxic, HIF-1α-transfected, and HIF-2α-transfected HEK293T cells, but not with extracts from normoxic HEK293T cells. This complex migrated to a position similar to that previously identified for hypoxic nuclear extracts with a 30-bp erythropoietin (EPO) oligo probe containing an HRE [26] (data not shown). HRE4 complex formation with HIF-1α and with HIF-2α was inhibited by competition with unlabeled wild-type HRE4 oligonucleotide at 50 and 100 times the concentration of labeled probe, but not by identical concentrations of mutant HRE4 oligonucleotides, suggesting that binding was specific to the HRE4 sequence (Figure 5). These data demonstrate that both HIF-1α and HIF-2α can bind to HRE4 of the PTPRZ1 promoter and that the HIF-2α activation specificity is not simply a function of differential binding of the HIFs to the HRE.

### ELK1 Is Required for Optimal HIF-2α Activation of the PTPRZ1 Promoter

The Ets transcription factor ELK1 has previously been reported to physically interact with HIF-2α protein, but not HIF-1α protein [13] and to facilitate the activation of CITED2, a gene whose response to hypoxia is primarily mediated by HIF-2α [13]. Knockdown of ELK1 by ELK1 siRNA significantly reduced hypoxic induction of CITED2. Similarly, interaction between HIF-2α and ETS1 was reported to be required for full transcriptional activation of FLK1gene, another HIF-2α-specific gene, in endothelial cells [14]. In another study, it was shown that HIF-2α cooperates with ETS1 transcription factor to activate the VE-Cadherin promoter [16].

To determine if specific activation of the PTPRZ1 promoter by HIF-2α might also involve an Ets, we examined the promoter sequence for putative Ets binding sites (EBS) using Genomatix software (Genomatix Software GmbH, Munchen, Germany). Several potential sites were identified, of which two, named here EBS4 (ggagcgaaGGAAatgttttt) and EBS5 (gaccgtctGGAAatgcgaatc) were present within the 250 bp promoter construct (Figure 1). Both contained the core Ets recognition motif 5′-GGA(A/T)-3′[27], shown in capital letters. EBS4 was located between HRE4 and HRE5 and EBS5 was located just downstream of HRE5. As ELK1 has previously been shown to bind to HIF-2 and be involved in hypoxic gene activation [13], we hypothesized that ELK1 might play a role in PTPRZ1 activation by HIF-2α.

**Figure 6:**
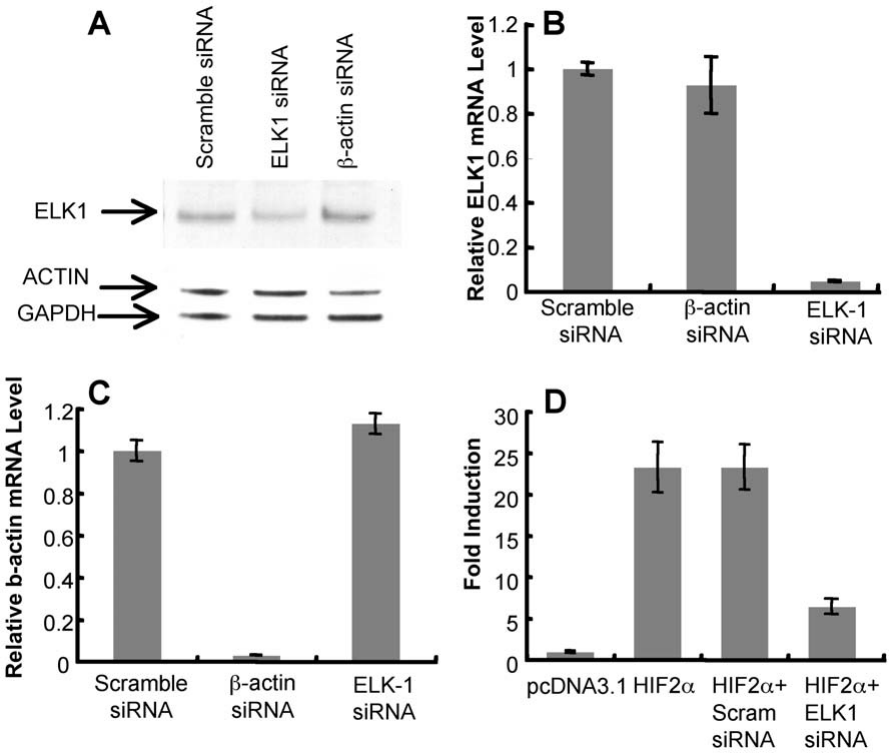
siRNA to ELK1 inhibits PRPRZ1 activation. (A) Immunoblotting analysis for ELK1, β-Actin, and GAPDH in HEK293T cells transfected with ELK1 scrambled siRNA (60 nM), β-Actin siRNA (60 nM), or ELK1 siRNA (60 nM). (B) ELK1 mRNA as determined by quantitative RT-PCR from HEK293T cells 48 hours after transfection with ELK1 scrambled siRNA, β-Actin siRNA, or ELK1 siRNA. (C) β-Actin mRNA under similar conditions. (D) PTPRZ1-250 promoter activity in HEK293T cells following transfection with HIF-2α plasmid alone or HIF-2α plasmid and either scrambled siRNA, or ELK1 siRNA. Data is presented as fold induction over vector control after normalization to β-gal. Bars represent mean and standard deviation of 3 determinations.

To test this hypothesis, siRNA experiments were performed on HEK293T cells using the PTPRZ1-250 bp promoter with a luciferase reporter. (Hep3B cells could not be utilized because of toxicity from the siRNA transfection reagent). Western blot analysis indicated that transfection of HEK293T cells with ELK1 siRNA resulted in a substantial reduction in ELK1 protein as compared to cells treated with scrambled siRNA or β-actin siRNA controls (Figure 6A). Also, real-time RT-PCR analysis indicated that relative levels of ELK1 mRNA decreased more than 90% following treatment with ELK1 siRNA (Figure 6B), and this was similar to the decrease observed for β-actin in cells treated with β-actin siRNA as a control (Figure 6C). When PTPRZ1-250-transfected HEK293T cells were co-transfected with HIF-2α in the presence of ELK1 siRNA, luciferase reporter activity decreased more than 75% as compared to cells transfected with HIF-2α alone or HIF-2α in combination with a scrambled siRNA control (Figure 6D) or to GAPDH siRNA control (results not shown). Taken together, these data suggest that ELK1 plays a role in the activation of PTPRZ1 by HIF-2α.

To explore this further, we assessed the effects of mutations in the EBS4 and EBS5 Ets binding sites. Four base-pair deletions (GGAA) were made in the Ets core recognition motifs of EBS4 or EBS5 within the 250 bp promoter construct and the activation by HIF-2α or HIF-1α transfection tested in the luciferase assay. The 4-bp deletion of the EBS4 core sequence decreased the activation of the promoter by HIF-2α by about half, as compared to the wild-type (WT) promoter sequence, while it had no appreciable effect on the activation by HIF-1α. By contrast, the 4-bp deletion of the EBS5 core sequence did not affect the promoter activation by HIF-2α or HIF-1α.

**Figure 7:**
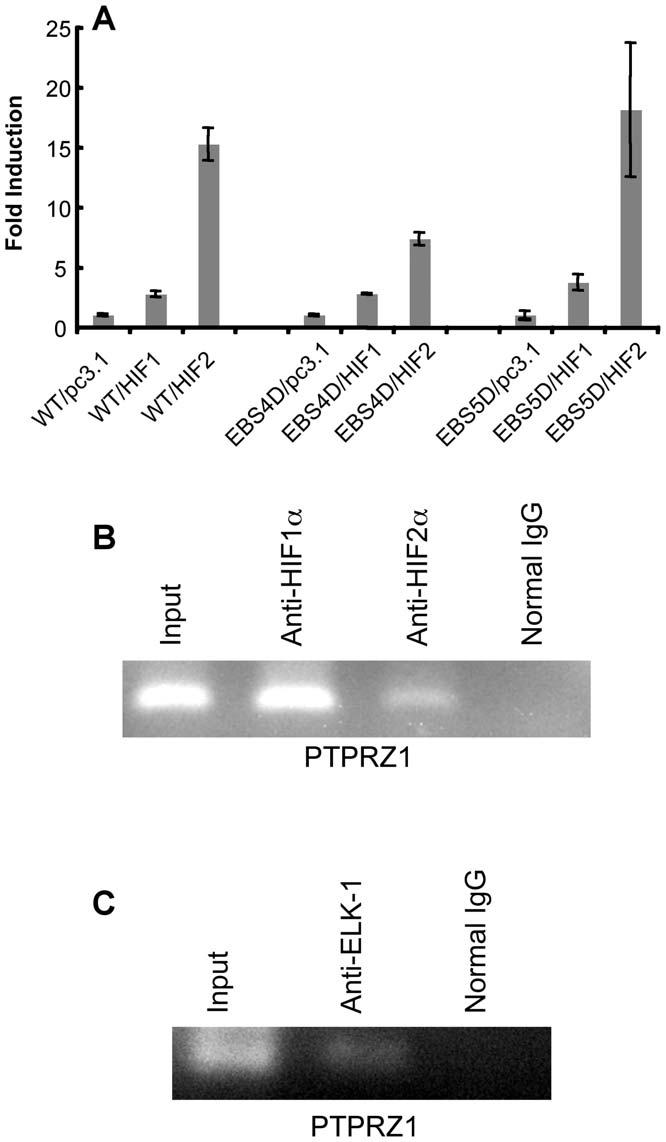
Role of EBS4 in the activation of PTPRZ1 promoter by HIF-2α. (A) Effects of EBS4 or EBS5 deletion on the response of the PTPRZ1 promoter to HIF-2α. HEK293T cells were co-transfected with 300 ng of PTPRZ1-250WT, EBS4D, or EBS5D promoters and 50 ng of a internal β-gal control plasmid in the presence of 250 ng of an expression plasmid encoding HIF-1α, HIF-2α or pcDNA3.1 empty control vector. Results are expressed as in Fig 2. (B). Binding of HIF-2α and HIF-1α to the PTPRZ1 promoter in the region near EBS4, EBS5, HRE4, and HRE5 *in vivo*. The chromatin immunoprecipitation assay was performed with HEK293T cells transfected with HIF-1α or HIF-2α respectively. Pre-cleared chromatin was immunoprecipitated with anti-HIF-2α or anti-HIF-1α antibody or normal rabbit IgG. After reversal of cross-linking, the DNA was analyzed by PCR. The primer set for PCR were designed to cover the EBS4, EBS5, HRE4, and HRE5 sites (C) Binding of ELK1 to the PTPRZ1 promoter. Experiment performed as in 7B except that the HEK293T cells were transfected with an Elk-1 expression vector and anti-ELK1 antibody or normal rabbit IgG was utilized.

To further assess the potential binding of HIF proteins and ELK1 in the region near EBS4, we utilized chromatin immonoprecipitation, amplifying the region that included HRE4, HRE5, EBS4, and EBS5. Using anti-HIF antibodies, we found that both HIF-1α and HIF-2α could bind in the promoter region of PTPRZ1 (Figure 7B). These results are consistent with the finding by electrophoretic mobility shift assay that either HIF-2α or HIF-1α could bind to HRE4. In addition, using anti-ELK1 antibody, we found that ELK1 bound in the same region. Collectively, these data provide evidence that ELK1 binds near HIF-2α and contributes to the induction of PTPRZ1 by HIF-2α.

## Discussion

In our initial study of the effects of HIFs on gene activation, PTPRZ1 was identified as one of 10 genes that were substantially upregulated by HIF-2α but not by HIF-1α [11]. In the current study, we sought to understand the molecular regulation of PTPRZ1 expression and in particular the basis for its HIF-2-specificity. In each of two cell lines studied, Hep3B (derived from human hepatoma cells) and HEK293T (derived from human embryonic kidney cells), substantially more PTPRZ1 promoter activity, as assessed using a luciferase reporter assay, was observed in response to HIF-2α as compared to HIF-1α. We found that much of the HIF-2-induced activation of PTPRZ1 could be attributed to HRE4, a near-tandem HRE on the antisense strand of the promoter region with the sequence TACGCACGCACGA. Furthermore, we found evidence that cooperative interaction between HIF-2α and the Ets factor ELK1 played a major role in mediating the HIF-2-specificity of PTPRZ1.

Since the discovery of HIF-2α [28], [29], researchers have explored the question of what role this seemingly redundant factor may have. Initial studies suggested that HIF-2 activated the same genes as HIF-1, but that its expression was largely limited to certain tissues and organs, particularly endothelial cells, renal tissue, lungs, heart, and small intestine [28], [29], [30]. More recent studies revealed that certain genes were preferentially or selectively activated by HIF-1 or HIF-2. Hu et al. found that hypoxic induction of several genes associated with glycolysis was selectively mediated by HIF-1 [12]. Our group and others subsequently found that a number of genes were preferentially upregulated by HIF-2, including CITED2, EPO, PAI-1, Tie2/TEK, VEGF receptor 2, and LOX [7], [11], [13], [14], [16].

This leads to the question of the mechanism for HIF-2 specificity. A number of functional HRE have been sequenced, and no obvious differences in the core HRE sequences between HIF-1 and HIF-2 target genes have emerged [4]. Our finding that the sequence of PTPRZ1 HRE4 is similar to other HREs and that this region bound to both HIF-1α and to HIF-2α is consistent with the concept that HIF-specificity is not mediated by the HRE core sequence. Several recent reports have provided evidence that HIF-2-specificity of certain genes can be conferred by cooperative binding of HIF-2α and members of the Ets family of transcription factors to elements in the genes' promoter regions [13], [14], [16], [31]. In particular, it was reported that the HIF-2-preferential activation of Flk-1, VE-cahedrin, and endosialin is mediated by an interaction between HIF-2α and ETS1, and that the HIF-2-preferential activation of CITED2 is mediated by an interaction between HIF-2α and ELK1 [13], [14], [16], [31]. We found that a 250 bp region of the PRPTZ1 promoter region that maintained HIF-2-specificity contained two putative Ets factor binding sites, that the HIF-2-responsiveness of this region could be impaired by siRNA to ELK1, that the HIF-2 responsiveness could be impaired by deletion mutation of one of the Ets binding sites (EBS4), and that HIF-2α and ELK1 could both bind to this region in proximity to each other. Also, ELK1 has previously been shown to physically interact with HIF-2α (but not HIF-1α) [13], and taken together, these results provide evidence that the HIF-2-specificity of PTPRZ1 is mediated at least in part by cooperative binding of HIF-2 and ELK1 to nearby sites in the PTPRZ1 promoter. It is possible that ELK1 binding to the promoter facilitates the binding of HIF-2α, or that these two factors work in concert to facilitate binding of another factor that provides optimal activation of PTPRZ1 transcription. These findings do not exclude a contribution from other Ets factors; due to the conservation of sequences within the ETS domain, Ets proteins all bind to similar DNA binding sequences, and the DNA sequence of EBS4 predicts it may also have binding to another Ets factor, ETS2 (references [27] and [32] and Genomatix Software). Nonetheless, the abrogation of the HIF-2α induction of PTPRZ1 by siRNA to ELK1 provides evidence that ELK1 plays an important role in the HIF-2α responsiveness.

It is noteworthy that in our original study using HEK293T cells, PTPRZ1 was activated by HIF-2α transfection but not by hypoxia [11]. This may in part reflect the fact that HEK293T cells exhibit relatively small increases of HIF-2 when exposed to hypoxia. At the same time, there is emerging evidence that stresses other than hypoxia may mediate increases in genes that are normally considered hypoxia responsive. For example, it has been reported that lysyl oxidase like 2 (LOXL2) is upregulated by high cell density through a mechanism that involves ETS1 [33]. Also, expression of HIF-2α, but not HIF-1α, has been observed in well oxygenated neuroblastoma tissue [34]. Interestingly, PTPRZ1 has recently been reported to be induced by chronic oxidative stress [35]. Perhaps related to this finding is the recent report that Sirtuin 1 (Sirt1), a deacetylase that is produced in response to redox stress, can enhance the activity of HIF-2α (but not HIF-1α) [5]. Thus, it is possible that under redox stress, Sirt1 enhances HIF-2α activity, which in turn promotes the overexpression of PTPRZ1.

PTPRZ1 is expressed predominantly in the central nervous system (CNS) during development and involved in signal transduction and recovery from demylenating lesions [19], [36]. In addition, PTPRZ1 and its ligand pleiotrophin are overexpressed in human glioblastoma [22] and there is substantial evidence that these proteins play an important role in the pathogenesis of this cancer (for review see [18]). Further evidence for the importance of PTPRZ1 in glioblastoma is provided by the finding that down regulation of PTPRZ1 by siRNA suppresses glioblastoma growth *in vitro* and *in vivo* [23]. Moreover, a monoclonal antibody to PTPRZ1 has been shown to delay tumor growth in a glioblastoma model [20]. There is also recent evidence that PTPRZ1 is upregulated and may play a role in the pathogenesis of a wide variety of tumors [20].

At the same time, there is increasing evidence that HIF-2 plays an important role in the pathogenesis of many tumors. Tumors require neovasculature to grow above a minimal size, or else they outstrip their oxygen supply, and HIFs stimulate the production of VEGF and other crucial vascular growth factors [6], [37]. Patients with VHL syndrome, who have increased levels of HIFs, develop a number of tumors including renal cell carcinoma [9]. Interestingly, there is evidence that tumorigenesis of renal cell carcinoma is associated with overexpression of HIF-2α, while HIF-1α inhibits tumor growth [10], [25], [38], [39]. Also, HIF-2α has recently been shown to enhance cell-cycle progression and tumorigenesis by promoting c-Myc transcriptional activity, while HIF-1α opposes c-Myc [15], [40].

The HIF-2-specific upregulation of PTPRZ1 may provide yet another mechanism by which HIF-2, but not HIF-1, promotes tumorigenesis in a variety of tumors. As noted above, while PTPRZ1 expression was initially thought to be largely limited to the central nervous system, it has been shown that PTPRZ1 is overexpressed in a variety of tumors, including hepatocarcinoma and renal carcinoma [20], [41], [42]. Also, PTPRZ1 has been shown to activate the β-catenin pathway, which can promote tumorigenesis [35], [43]. The potential role of HIF-2 upregulation of PTPRZ1 in renal cell carcinoma of particularly intriguing, as VHL is associated with renal cell carcinoma and there is evidence that HIF-2 is more important than HIF-1 in the renal cell carcinoma tumorigenesis in this setting [38]. Taken together, these results suggest that activation of PTPRZ1 by HIF-2α and ELK1 may be an important step in the pathogenesis of a variety of tumors. Further study of these relationships may help inform strategies to prevent and treat these tumors.

## Material and Methods

### Cell Culture

HEK293T and Hep3B cell lines were obtained from ATCC (American Type Culture Collection, Manassas, VA)where they were authenticated by short tandem repeats and karyotyping, and frozen in liquid nitrogen. After thawing, the cell lines were maintained in Dulbecco's modified Eagle's medium supplemented with 10% fetal bovine serum (Heat inactivated, Hyclone, Logan, Utah) and penstrep/glutamine (Invitrogen Corp, Carlsbad, CA), and used within 6 months. Cells were incubated in 95% air and 5% CO2 (normoxia) or 1% O2 and 5% CO2 (hypoxia) at 37°C in a standard tissue culture incubator.

### Plasmid DNA Construction and Site-Directed Mutagenesis

Expression plasmids encoding human HIF-1α (pHAHIF1α-pcDNA3) and a degradation-resistant form of HIF-1α (drHIF-1α) were gifts from Eric Huang (NCI, NIH) [44]. An expression plasmid encoding human HIF-2α (hEPAS1-pcDNA3) was a gift from Steven L. McKnight (University of Texas) [28]. A plasmid encoding a degradation-resistant form of HIF-2α (drHIF-2α) was created by mutating prolines 405 and 531 of HIF-2α to alanine. All plasmids were purified with a Qiagen Maxiprep kit (Qiagen, Valencia, CA), and inserts were verified by restriction enzyme mapping and DNA sequencing. PTPRZ1 gene promoter luciferase reporter constructs were created containing regions spanning nucleotides −2138bp, −1655bp, or −1059bp upstream to +57bp exon I (Figure 1). DNA fragments were amplified from human genomic DNA (Cat#G304A Promega Madison WI) by PCR with 5′primers PTF1, PTF2, PTF3 ( 5′-CAGTGGTACCGTTTGTCACAGTCTGACCC-3′, 5′-CAGTGGTACCCTGCCATTAGCTTGCACA-3′, 5′-GAGCGGTACCGCATACCCTTCAATCAAGC-3′) and 3′primer PTR2( 5′-TAGTAAGATCTACCGATCCCGGAGCTGAA-3′) which contain Kpn1/BglII sites. PCR fragments were purified and cloned into the corresponding sites of the luciferase reporter vector pGL3basic (Promega) to generate three different PTPRZ1 promoter reporter plasmids (Figure 1). A PTPRZ1-250 promoter was created and amplified by PCR using PTPRZ1-1655 plasmid DNA as template with 5′primers PTFc (5′-AGTCATGCTAGCGTGCGGCTTTCTCCAGAT-3′) and 3′primer PTR3 (5′-ATGATACTCGAGTTCCAGACGGTCTGCGGC-3′), which contain NheI/XhoI sites. This PCR fragment ends before the ATG. PCR fragments were inserted into the corresponding sites of pGL3basic. Plasmid DNAs PTPRZ1-2138M4, -1655M4, -1059M4 and -250M4 expressing a mutagenized HRE (HRE4) were constructed using PCR-based QuickChange site-directed mutagenesis kit (Stratagene, La Jolla, CA). The primers contain the mutated HRE4 sequences, 5′ primer ( 5′-CACACAAACACACATACGCGCATACGATCTCACTTCGATC-3′) and complementary 3′ primer ( 5′-GATCGAAGTGAGATCGTATGCGCGTATGTGTGTTTGTGTG-3′). This mutant plasmid DNA contained a 3 nucleotide substitution (underlined) in the HRE4 element (from GCACGCACG to GCGCATACG). Reactions were performed for 16 cycles at 95°C for 30 seconds, 55°C for 1 min and 68°C for 7 min. PCR products were incubated with DpnI enzyme at 37°C for 2 hrs to digest the parental vector. DNA was used to transform XL1-Blue competent cells. Constructs were confirmed by DNA sequencing. Plasmid DNA PTPRZ1-2138M5, -1655M5, -1059M5 and -250M5 with a mutagenized HRE5 were generated by using the same technique and primer set sequences as follows: 5′ primer (5′-GAAGCAGAGGAGCCGCTTTGCGAGGGGCCGCAGA-3′), and 3′ primer (5′-TCTGCGGCCCCTCGCAAAGCGGCTCCTCTGCTTC-3′) (HRE5 wild type sequences were mutated from GCACG to GCTTT). Plasmid DNA PTPRZ1-EBS4D and EBS5D with deletion of Ets-binding core motifs GGAA were generated by using the same technique and primer set sequences as follows: 5′ primer (5′ CAAAAAAAACATTTCGCTCCCCCTCCCTCTCC 3′), and 3′ primer (5′ GGAGAGGGAGGGGGAGCGAAATGTTTTTTTTG 3′). EBS5D primer sets were: 5′ primer (5′ ACGGCGAGGGGCCGCACTCGAGATCTGCGATCT 3′), and 3′ primer (5′ AGATCGCAGATCTCGAGTGCGGCCCCTCGCCGT 3′).

### Transfection and Luciferase Reporter Assays

Cells were transfected as described [11] except that Fugene6 transfection reagent (Roche, Indianapolis, IN) was utilized. For luciferase reporter experiments, 1.5×10^5^ of HEK293T cells or 0.5×10^5^ Hep3B cells/well were plated in a 12 well plate and the following day were co-transfected with 300 ng/well of reporter plasmid DNA and 250 ng/well of a HIF or a control (pcDNA3.1) expression plasmid in the presence of 50 ng of an internal control plasmid DNA, pSV-β-gal (Promega) to normalize transfection efficiency. After transfection, cells were incubated in normoxia for 48 hrs. Cells were then washed with phosphate buffered saline (PBS), lysed with 250 µl per well of 1x reporter lysis buffer (Promega), and freeze-thawed once. After samples were centrifuged at 13,000×g for 5 min, 20 µl and 50 µl of cell lysates were used to determine luciferase and β-gal in the extracts. The expression vector pcDNA3.1 was set at unity and the values for different promoters compared to this control as fold induction.

### Electrophoretic Mobility Shift Assay

Nuclear extracts were prepared as described [45]. Electrophoretic mobility shift assay was performed using a digoxigenin (DIG) gel shift kit (Roche Applied Science, Indianapolis, IN). Synthetic oligonucleotides were annealed with their complementary strand and labeled with DIG at the 3′end[26]. Labeled DNA probes were incubated with 7.5 µg of nuclear extract for 20 min at 4°C. Poly(dI-dC) was added to reduce nonspecific binding. For competition assays, labeled probes were incubated with increasing amounts of cold unlabeled wild type or mutant double-stranded oligonucleotides (50x, 100x) and nuclear extract. Protein-DNA complexes were separated on a 6% non-denaturing polyacrylamide gel in 0.5x Tris-borate-EDTA buffer, transferred to nylon membrane, and probed with 1:15,000 dilution of antidigoxigenin antibody conjugated to alkaline phosphatase (Roche Applied Science, Indianapolis, IN). Bands were visualized by Western Blue stabilized substrate (Promega). Gels were scanned using the Epson Perfection 4990 Scanner (Epson America, Long Beach, CA) and processed using Silverfast Launcher Software V2.1.3 (LaserSoft Imaging, Sarasota, FL). Figures were prepared for publication using Adobe Photoshop CS, Version 8.0 for the Macinosh.

### siRNA Transfection

siRNA duplexes targeting ELK1 were purchased from Ambion (Austin, TX). Silencer negative control siRNA and siRNA for β-actin as a positive control were used. HEK293T cells at 50–60% confluence were transfected with siRNA duplexes at a final concentration of 60 nM in complete DMEM (Invitrogen) using 3 µl of siPORT Amine transfection reagent (Ambion). The mixture of siRNA, plasmid DNAs and transfection reagent were prepared in 200 µl of serum-free DMEM. The first transfection mixture contained siRNA, plasmid DNAs and transfection reagent. Twenty-four hrs later, the transfection procedure was repeated without plasmid DNAs, and cells were incubated for another 48 hrs in normoxia and cell lysates were prepared.

### Immunoblotting

Whole cell lysates were prepared from HEK293T cells using an M-PER extraction kit (Pierce, Rockford, IL). Five to 20 µg of lysate were electrophoresed on pre-cast 4–12% Tris-Bis NuPAGE (Invitrogen) and transferred to nitrocellulose. The membrane was blocked with 5% w/v nonfat dry milk in 1xTBST(10 mM Tris-Hcl, pH 8.0, 150 mM NaCl, and 0.05% Tween 20) overnight at 40°C. Blots were then incubated with monoclonal antibody to GADPH or to β-actin, or rabbit polyclonal antibody to ELK1 (Santa Cruz Biotechnology, Santa Cruz, CA). After 1 hr incubation with appropriate secondary antibody conjugated to alkaline phosphatase (Promega), bands were visualized with Western Blue stabilized substrate (Promega). Membranes were scanned and the images processed as described above.

### Quantitative Real-Time PCR

Total RNA was extracted from HEK293T cells 48 hrs post transfection with RNAeasy kit (Qiagen, Valencia, CA). Five µg each of total RNA was treated with RNase free DNaseI (Ambion) [11]. First-strand cDNA was synthesized from 820 ng of total RNA using Roche First Strand cDNA Synthesis kit (Roche Applied Science). For real-time PCR experiments, 41 ng of RNA (start) was amplified in 2xSYBR PCR master mix using TF-Stepone RT PCR System (PE applied Biosystems, Branchburg, NJ) with the following primers (shown in 5′ to 3′ direction): for Actin, CCTTCCTGGGCATGGAGT and CAGGGCAGTGATCTCCTTCT; for GAPDH, CCAGCCGAGCCACATCGCTC and ATGAGCCCCAGCCTTCTCCAT; for ELK1, CCTGTCTGCGTTTTTGGATGTG and TGTCTGAGAGAAAGGTTGGGGG. Samples were incubated at 95°C for 10 minutes, followed by 40 cycles, each consisting of 95°C for 15 seconds, 60°C for 1 minute. Melting curves were generated after each run to confirm amplification of specific transcripts. All quantifications were normalized to human β-actin. Standard curves were obtained for each primer pair with serial dilutions of cDNA templates. Real time PCR reactions were performed in triplicate and each experiment repeated three times.

### Chromatin Immunoprecipitation Assay

Chromatin immunoprecipitation assay was performed using the EZ-Magna chip™ A chromatin immunoprecipitation kit (Millipore, USA) following manufacturer′s instructions. Briefly, 293T cells (10^6^) were transfected with HIF-2α, HIF-1α, or Elk-1 expression vectors (GeneCopoeia Inc. Rockville MD). At 36 h post-transfection, cells were cross-linked with formaldehyde at a final concentration of 1% at room temperature for 10 min. The unreacted formaldehyde was quenched using 2M glycine for 5 min at room temperature before harvest. Cells were scraped and resuspended in lysis buffer containing protease inhibitors. Soluble chromatin was prepared after sonication with a Misonix XL-2000 sonicator (Osonica, LLC, Newtown, CT, USA) to an average DNA length of 200 to 1000 bp. Approximately 3×10^5^ cell- equivalents of the pre-cleared chromatin was immunoprecipitated with 5 µg of anti-HIF1α, anti-HIF2α (Novus Biologicals), anti-Elk-1 or normal IgG (Santa Cruz Biotechnology) antibody together with protein A magnetic beads for overnight at 4°C on a rotating platform. 10% of the pre-cleared lysate was set aside as input control. Immune complexes were washed with high salt immune complex buffer, low salt immune complex buffer, lithium chloride immune complex buffer, and TE buffer each for 4 min at room temperature on a rotating platform. The immune complexes were eluted and the DNA-protein cross-linking was reversed by heating at 62°C for 2 h with constant shaking. The chromatin was purified using spin columns provided with the kit and analyzed by PCR using the specific primers (PTPRZ1: 5′-TCC AGA TTA TTC CTC TCT CGC-3′, 5′-AGA GGA GCT GAA TGC AAG CG-3′); the region spanned by these primers includes HRE4, HRE5, EBS4, and EBS5. PCR products were electrophoresed with a 2% agarose gel stained with ethidium bromide.
